# Hold out Your Tongue

**Published:** 1895-07

**Authors:** 


**"Hold Out Your Tongue."**—My doctor is a real joker," said a Lewiston lady. "I didn't know that my talking bothered him, when he was writing prescriptions, until yesterday. He never mentioned it, and I always asked him all sorts of questions while he was writing them out. Yesterday he examined me, and sat down to write something. I kept talking. Suddenly he looked up, and said: 'How has your system been? Hold out your tongue.' I put out that member, and he began to write. He wrote, and I held out my tongue, and when he got through he said: 'That will do.' 'But,' said I, 'you haven't looked at it.' 'No,' said he, 'I don't care to, I only wanted to keep it still while I wrote the prescription.' "—*Ex.*

